# Supplementary material for: Granuloma dual RNA-seq reveals composite transcriptional programs driven by neutrophils and necrosis within tuberculous granulomas
Source: Sci Adv. 2026 Jan 21;12(4):eadw4619. doi: 10.1126/sciadv.adw4619 (PMC12822655; doi:10.1126/sciadv.adw4619)
Supplement: Supplementary file 1 — Figs. S1 to S11 Legends for tables S1 to S10 Legends for movies S1 to S7 [file sciadv.adw4619_sm.pdf]

Science Advances
AAAS

Supplementary Materials for

Granuloma dual RNA-seq reveals composite transcriptional programs driven by neutrophils and necrosis within tuberculous granulomas

Gopinath Viswanathan *et al.*

Corresponding author: Gopinath Viswanathan, gopinath.viswanathan@duke.edu; David M. Tobin, david.tobin@duke.edu

Sci. Adv. **12**, eadw4619 (2026)
DOI: 10.1126/sciadv.adw4619

The PDF file includes:

Figs. S1 to S11
Legends for tables S1 to S10
Legends for movies S1 to S7

Other Supplementary Material for this manuscript includes the following:

Tables S1 to S10
Movies S1 to S7

Fig. S1

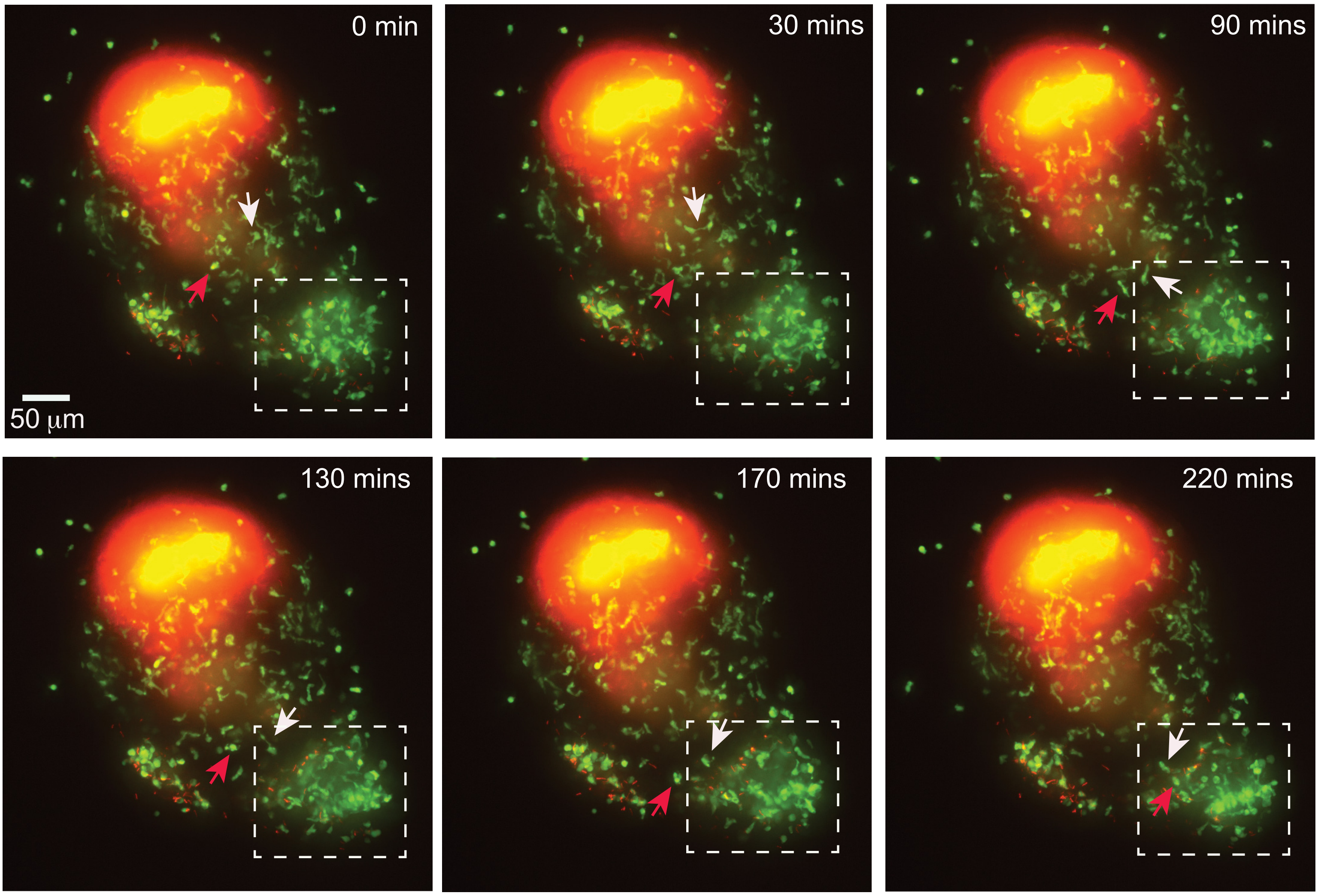

Fig. S1. Neutrophils migrate between sub-compartments of a type II granuloma. Movement of two neutrophils tracked over 220 mins (red and white arrows) from a region near the necrotic core to the ENR (white dashed box) of a type II granuloma. Scale bar, 50 μm.

Fig. S2

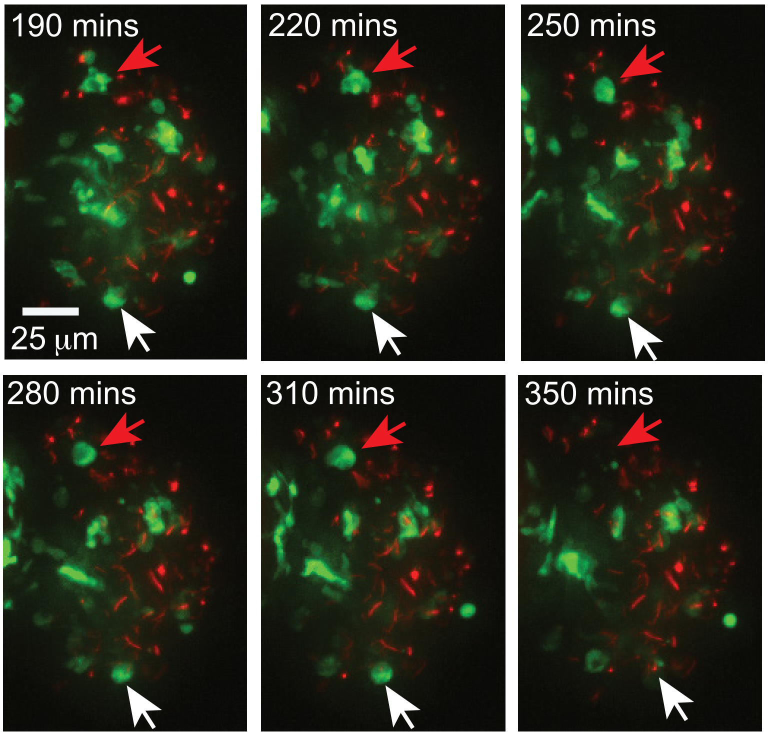

Fig. S2. Neutrophil death in type II granulomas is independent of the cell infection status. Magnified images of the extra–necrotic region (ENR) of a type II granuloma shown in **Fig. 1G** at different time points. The red arrow indicates an uninfected neutrophil; the white arrow indicates an infected neutrophil. Scale bar, 25 µm.

Fig. S3

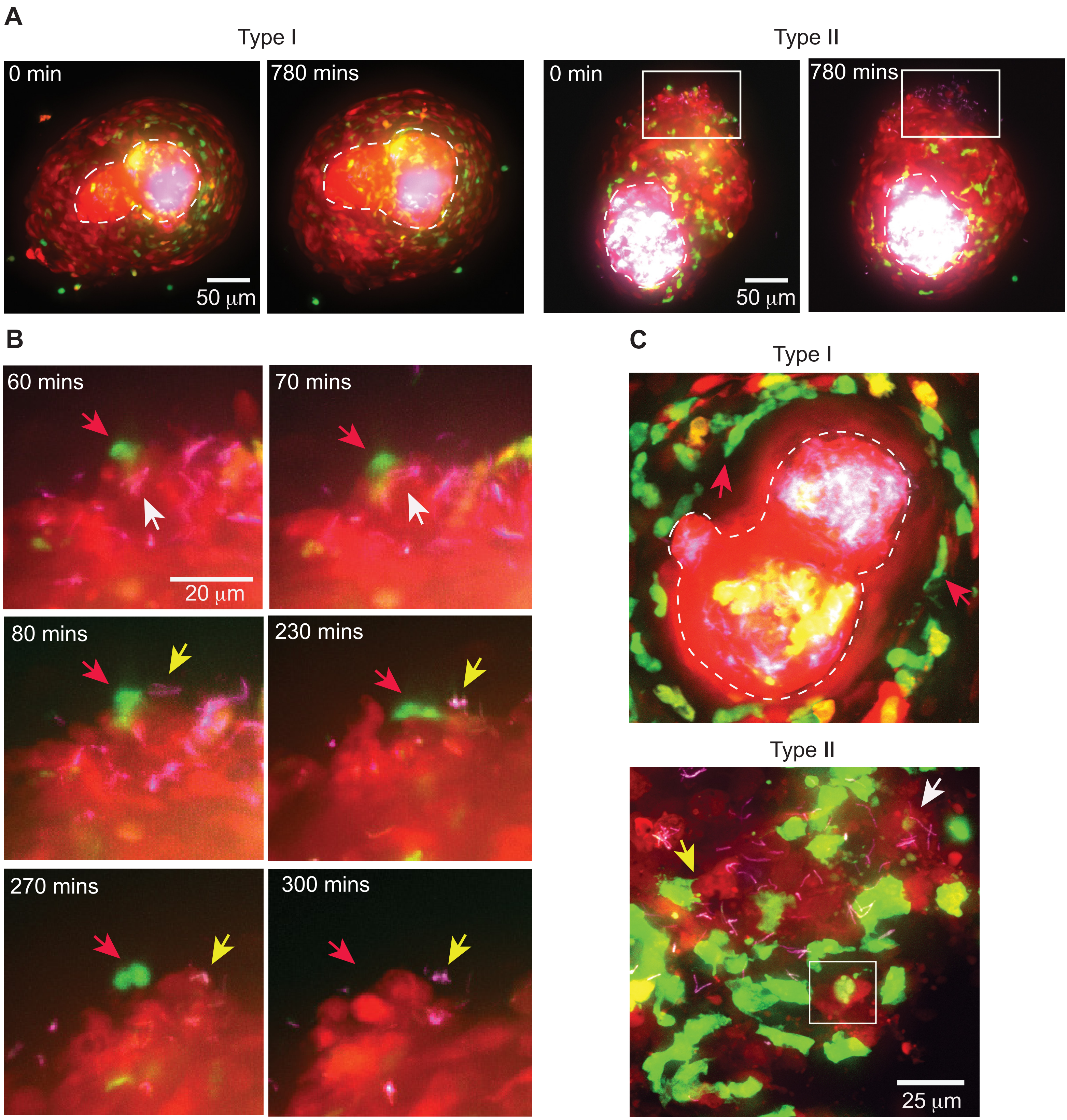
A
Type I
0 min
780 mins
50 μm
Type II
0 min
780 mins
50 μm
B
60 mins
70 mins
20 μm
80 mins
230 mins
270 mins
300 mins
C
Type I
Type II
25 μm

Fig. S3. Granuloma microenvironment governs neutrophil macrophage interactions. (A) Fluorescent time-lapse images showing macrophage (red) and neutrophil (green) viability in *M. marinum* (magenta) - infected type I and ENR (white box) of type II granulomas observed over 780 mins. Images are representative of granulomas from three independent experiments, n = 9 animals, scale bar, 50 µm. **(B)** Magnified images of the ENR (white boxes in panel A) at different time points, showing interactions between a dying neutrophil (red arrow) and an infected macrophage (white arrow). The yellow arrow indicates *M. marinum* released from the infected macrophage after its interaction with the neutrophil. Scale bar, 20 µm. Fluorescent images are 100 µm maximum projections. **(C)** Representative fluorescent images of tissue sections showing neutrophil morphologies and their interactions with macrophages in granuloma subtypes. Red arrows indicate elongated neutrophils in a type I granuloma. In the ENR of type II granuloma, the yellow arrow shows the interaction between a neutrophil and an infected macrophage, the white arrow shows an infected macrophage containing phagocytosed neutrophil debris, and the white box shows a macrophage engulfing a whole neutrophil. Fluorescent images are 20 µm maximum projections. Images are representative of tissue sections from 2 animals, scale bar, 25 µm. Boundaries of the necrotic cores in fluorescent images are marked with white dashed lines.

Fig. S4

A

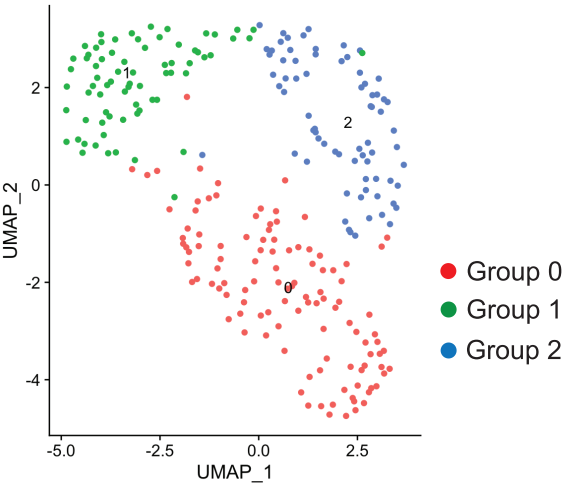

B

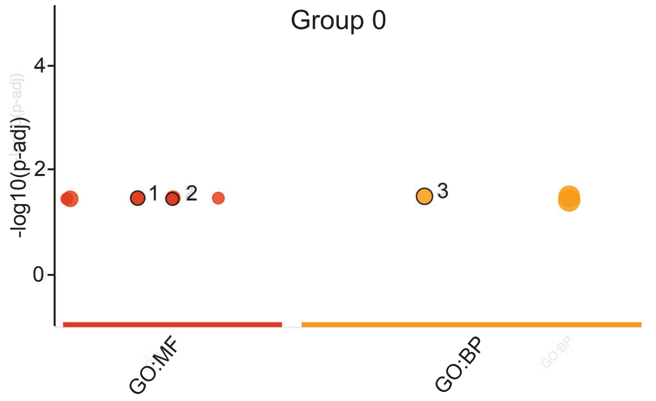

id	source	term_id	term_name	term_size		p_value
1	GO:MF	GO:0023026	MHC class II protein complex binding	7		3.5e-02
2	GO:MF	GO:0042287	MHC protein binding	5		3.6e-02
3	GO:BP	GO:0019882	antigen processing and presentation	12		3.2e-02

C

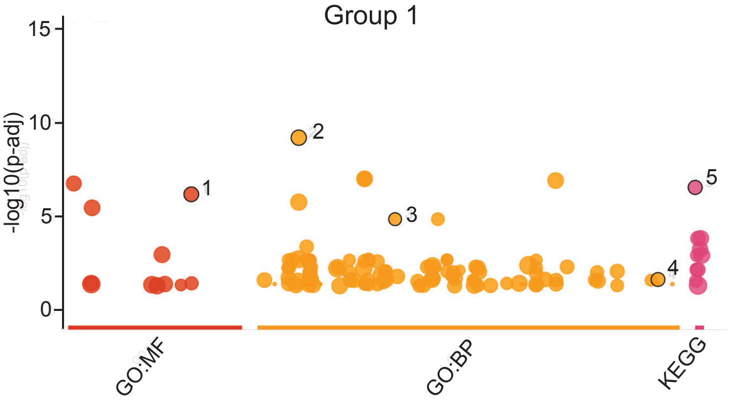

id	source	term_id	term_name	term_size		p_value
1	GO:MF	GO:0051015	actin filament binding	93		6.7e-07
2	GO:BP	GO:0007015	actin filament organization	152		6.7e-10
3	GO:BP	GO:0034314	Arp2/3 complex-mediated actin nucleation	23		1.4e-05
4	GO:BP	GO:1990266	neutrophil migration	46		2.2e-02
5	KEGG	KEGG:00010	Glycolysis / Gluconeogenesis	56		2.9e-07

D

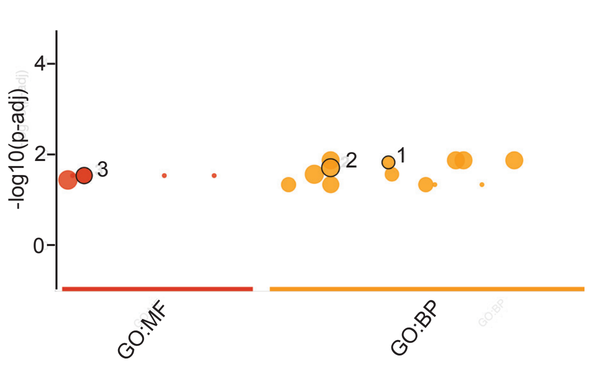

id	source	term_id	term_name	term_size		p_value
1	GO:BP	GO:0030223	neutrophil differentiation	14		1.5e-02
2	GO:BP	GO:0009605	response to external stimulus	638		2.0e-02
3	GO:MF	GO:0005125	cytokine activity	149		3.0e-02

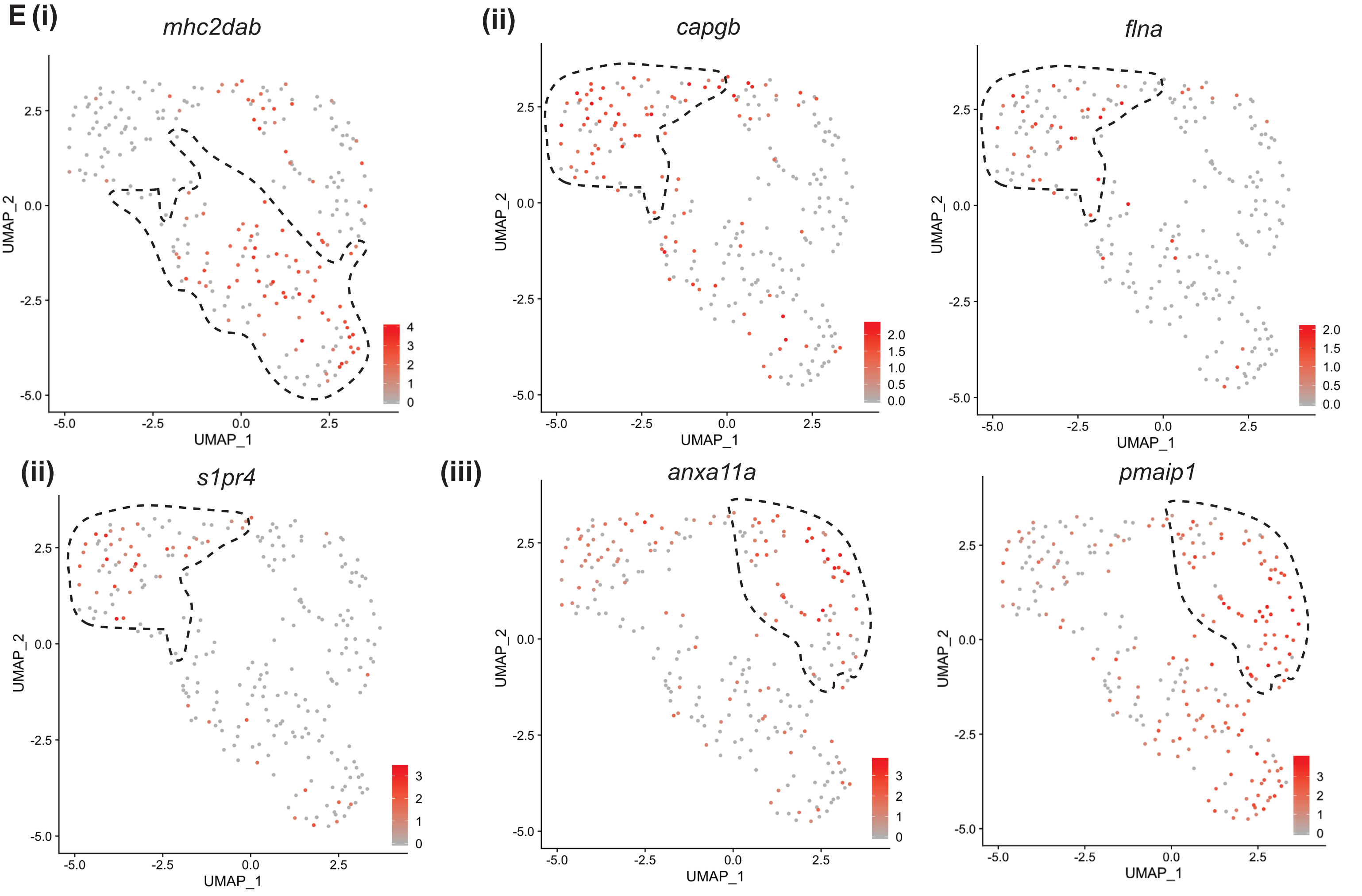

Fig. S4. scRNA-seq analysis of mature granulomas reveals neutrophil subgroups with distinct transcript profiles. (A) UMAP plot of scRNA-seq data of neutrophils from micro-dissected granulomas. (**B-D)** Manhattan plots depicting gene ontology (GO) analysis of transcript profiles from the neutrophil subgroups shown in **(A).** GO terms relevant to this study are highlighted and numbered. MF – Molecular Function, BP – Biological Process, KEGG - Kyoto Encyclopedia of Genes and Genomes **(E)** Expression maps of representative genes in neutrophil subgroups, with the corresponding neutrophil subgroups marked by black dashed lines. **(B-D)** False Discovery Rate (FDR) correction was used to determine the adjusted p-values.

Fig. S5

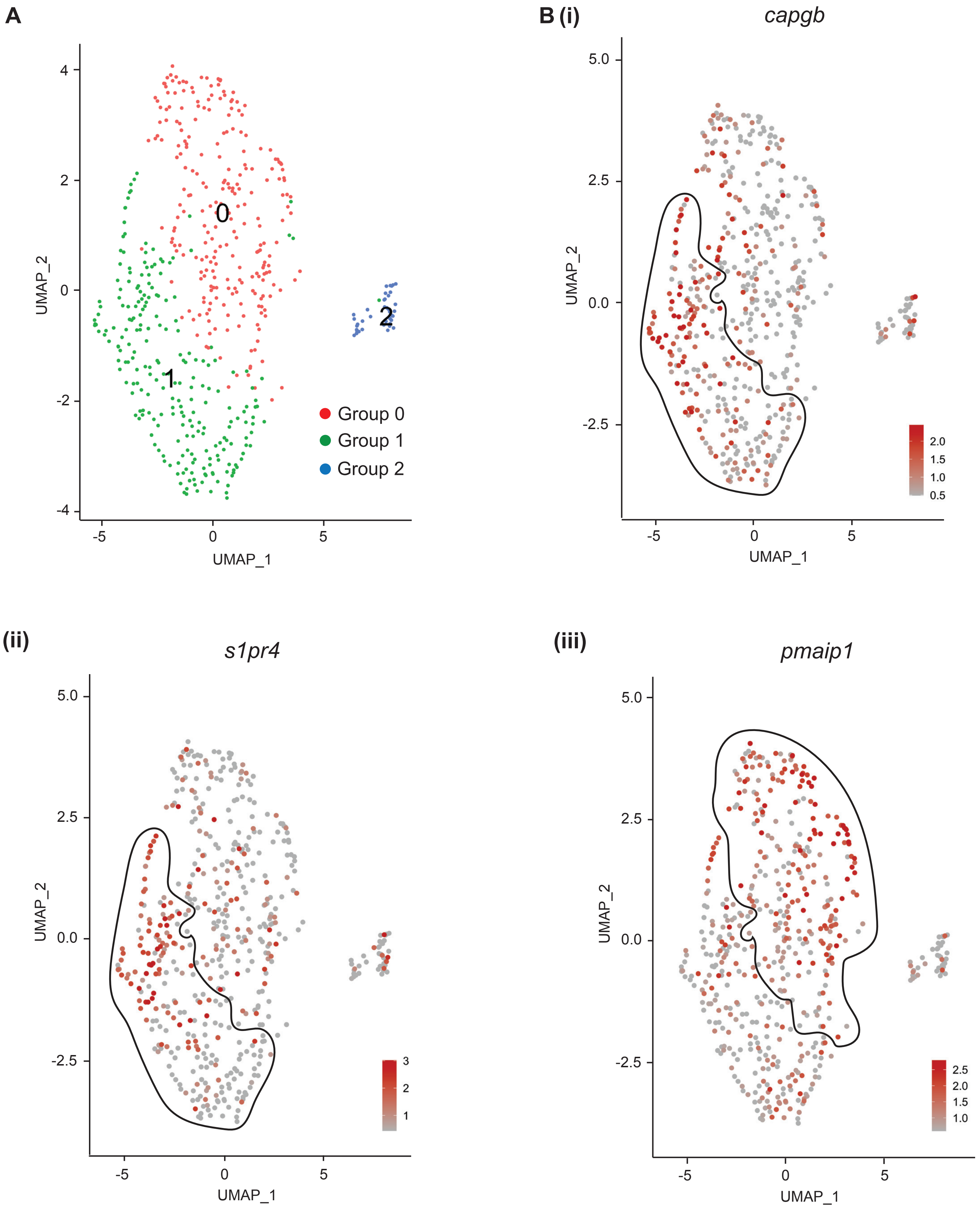
A
Group 0
Group 1
Group 2
UMAP_1
UMAP_2
B (i)
capgb
(ii)
s1pr4
(iii)
pmaip1

Fig. S5. Replicate scRNA-seq analysis showing consistent neutrophil subgroup transcript profiles in mature granulomas. (A) UMAP plot of scRNA-seq data showing neutrophils from micro-dissected granulomas. The data represent the second independent experiment corresponding to Fig. S4. **(B)** Expression maps of representative genes from Fig. S4E, with the corresponding neutrophil subgroups outlined.

Fig. S6

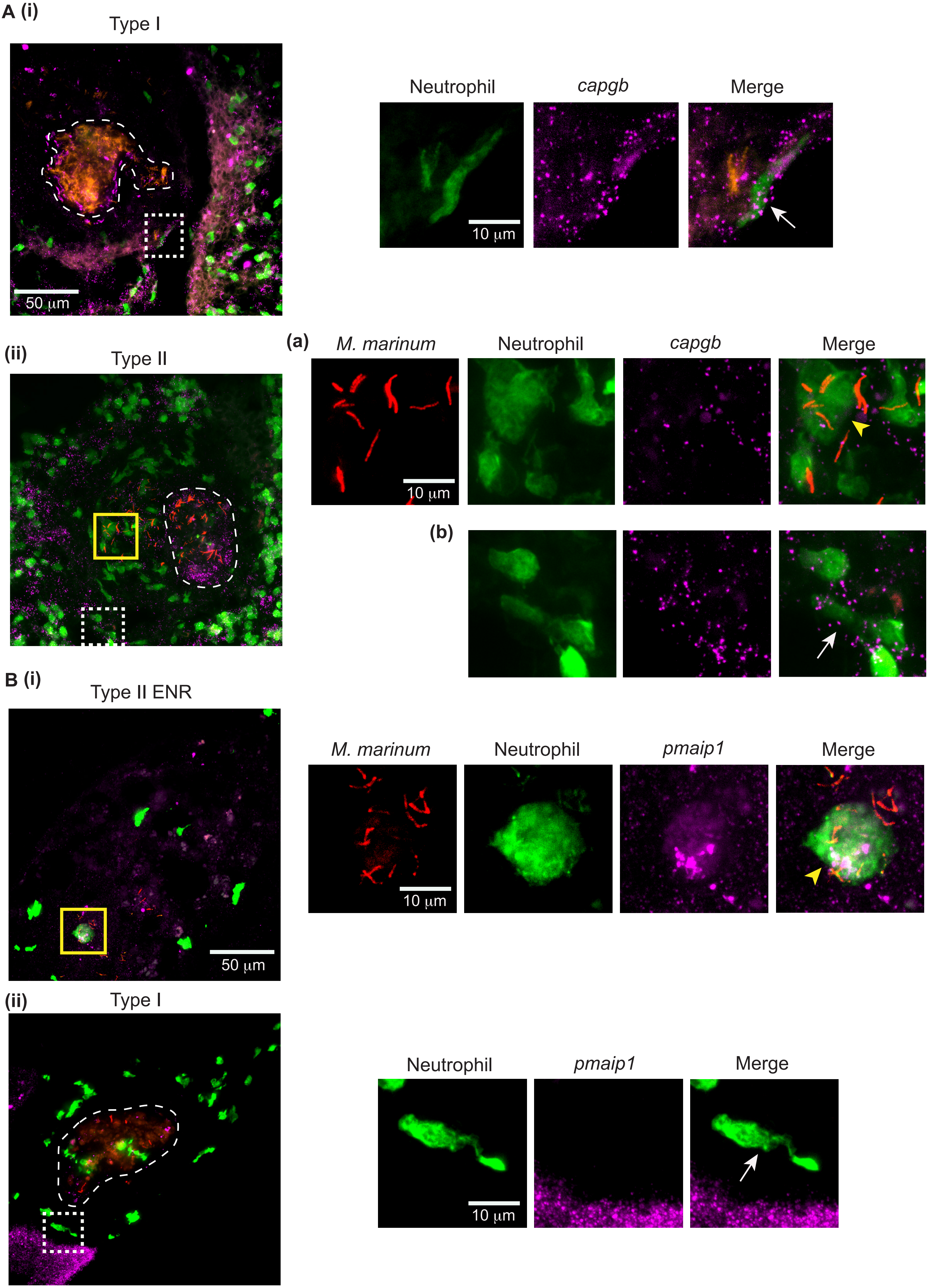

A (i)
Type I
50 µm
Neutrophil
capgb
Merge
10 µm
(ii)
Type II
(a)
M. marinum
Neutrophil
capgb
Merge
10 µm
(b)
B (i)
Type II ENR
50 µm
M. marinum
Neutrophil
pmaip1
Merge
10 µm
(ii)
Type I
Neutrophil
pmaip1
Merge
10 µm

Fig. S6. Detection of transcripts enriched in granuloma neutrophil subtypes by HCR RNA-FISH. (A) i & ii: Representative fluorescent images of adult zebrafish tissue sections showing enrichment of *capgb* mRNA (magenta puncta) in elongated neutrophils (dotted white boxes in the main panels and white arrows in the insets) of **(i)** Type I and **(ii)** Type II granulomas, compared to the rounded neutrophils (yellow arrowhead in the inset) in the ENR (yellow box) of **(ii)** Type II granulomas. **(B) i & ii:** Representative fluorescent images showing *pmaip1* mRNA (magenta puncta) in a rounded neutrophil (yellow box/yellow arrowhead) of **(i)** Type II ENR, compared with an elongated neutrophil (dotted white box/white arrow) of **(ii)** Type I granuloma. **(A, B)** Neutrophils are labelled green and *M. marinum* in red. Boundaries of the necrotic cores are marked with white dashed lines. Scale bars: main panels, 50 µm; insets, 10 µm. Images are representative of tissue sections from two animals.

Fig. S7

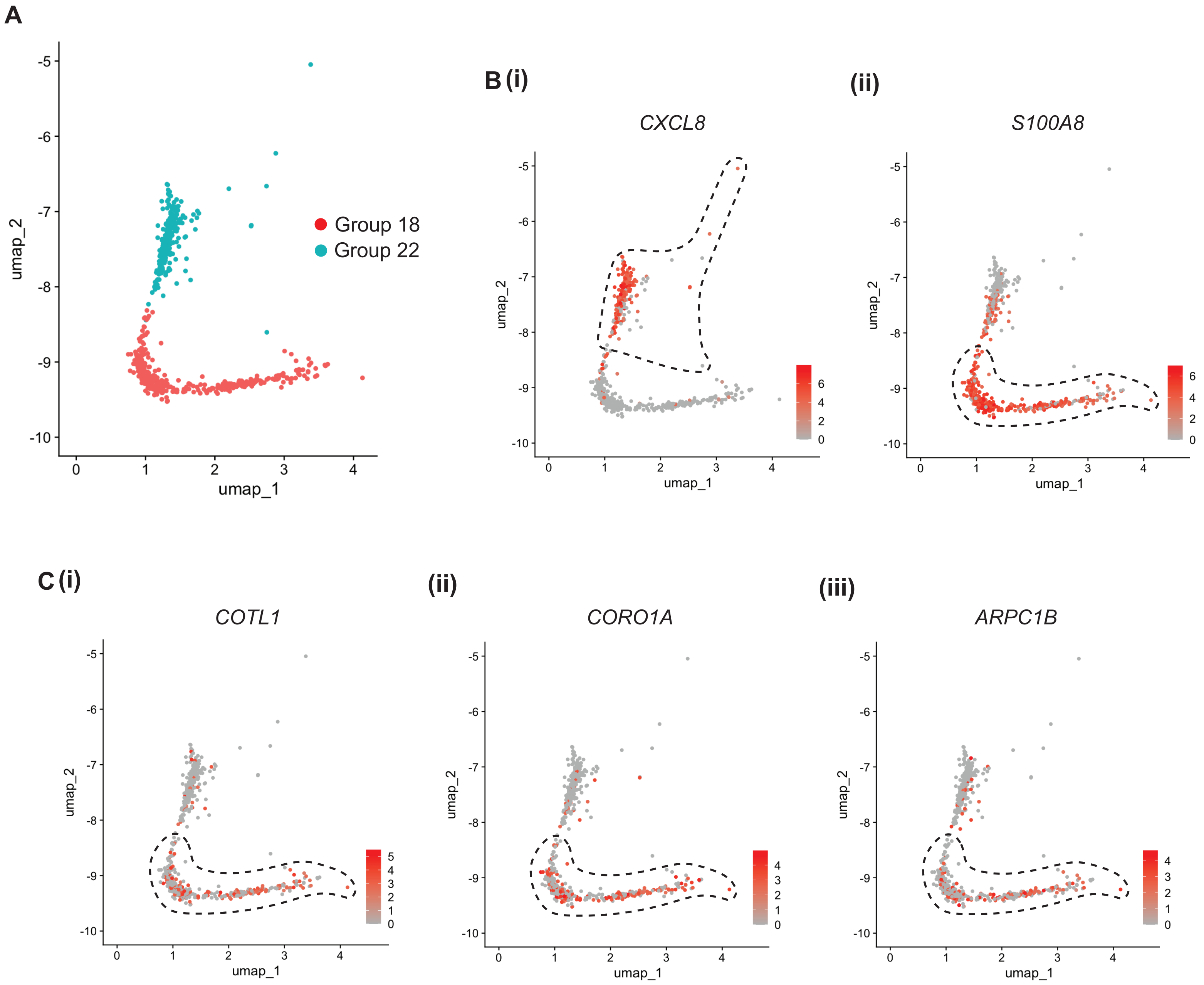

A
Group 18
Group 22
umap_1
umap_2
B (i)
CXCL8
(ii)
S100A8
C (i)
COTL1
(ii)
CORO1A
(iii)
ARPC1B

Fig. S7. scRNA-seq analysis of human pulmonary granulomas reveals a neutrophil subcluster enriched with actin-associated transcripts. **(A)** UMAP plot of scRNA-seq data generated from tissue of human TB granuloma biopsy specimens showing neutrophil subclusters. **(B)** Transcriptional signatures defining each neutrophil sub-cluster. **(C)** Expression maps of actin-associated transcripts with the corresponding neutrophil sub-cluster marked by black dashed lines.

Fig. S8

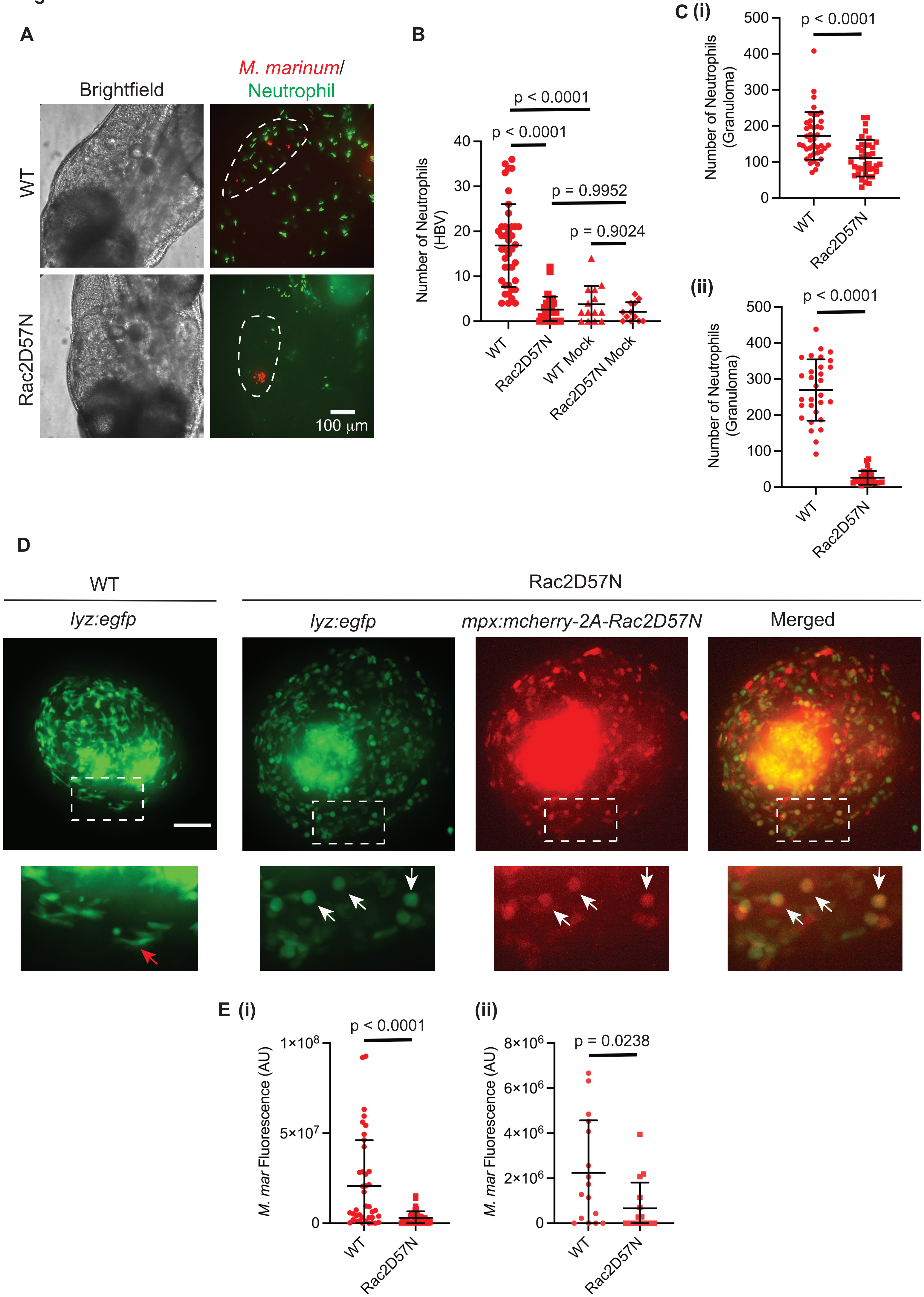
A
Brightfield
M. marinum/
Neutrophil
WT
Rac2D57N
100 µm
B
Number of Neutrophils (HBV)
p < 0.0001
p < 0.0001
p = 0.9952
p = 0.9024
WT
Rac2D57N
WT Mock
Rac2D57N Mock
C (i)
Number of Neutrophils (Granuloma)
p < 0.0001
(ii)
p < 0.0001
D
WT
Rac2D57N
lyz:egfp
lyz:egfp
mpx:mcherry-2A-Rac2D57N
Merged
E (i)
M. mar Fluorescence (AU)
p < 0.0001
(ii)
p = 0.0238

Fig. S8. Characterization of Rac2D57N fish during *M. marinum* infection. (A) Representative bright-field and fluorescent images of WT and Rac2D57N larval fish showing neutrophil (green) recruitment to the hindbrain ventricle (outlined with dotted lines) at 3 dpi with *M .marinum* (red). Scale bar, 100 µm. Fluorescent images are maximum projections spanning the entire hindbrain ventricle. **(B)** Mean number of neutrophils recruited to the hindbrain ventricle of WT and Rac2D57N larvae following mock or *M .marinum* infection (3dpi). Each data point represents the number of neutrophils recruited to the hindbrain ventricle of a single larva. WT: 41 fish; Rac2D57N: 36 fish; WT mock: 14 fish; Rac2D57N mock: 12 fish. One-way ANOVA with Tukey's multiple comparison test was used. **(C) i & ii:** Mean number of neutrophils observed in the Rac2D57N granulomas and their WT siblings. Each data point represents the number of neutrophils observed in a single granuloma. The data shows the remaining two independent experiments corresponding to Fig. 2I. **i)** n = 41 granulomas for WT & 37 granulomas for the Rac2D57N, obtained from 3 animals each; **ii)** n = 30 granulomas for both WT and Rac2D57N, obtained from 3 animals each. **i)** Two-tailed, unpaired t-test; **ii)** Unpaired t-test with Welch's correction. **(D)** Fluorescent images of granuloma explants showing altered neutrophil morphologies in Rac2D57N-expressing neutrophils (white arrows in the insets) compared to a WT neutrophil (red arrow). Scale bar, 50 µm. **(E) i & ii:** *M. marinum* burden in WT and Rac2D57N granulomas represented as mean arbitrary fluorescence units (AU). Granulomas were dissected from 14 dpi animals. Each data point represents *M. marinum* fluorescence measured in a single granuloma. The data shows the remaining two independent experiments corresponding to Fig. 2K. **i)** n = 40 granulomas for WT & 37 granulomas for the Rac2D57N, obtained from 3 animals each; **ii)** n = 16 granulomas for both WT and Rac2D57N, obtained from 3 animals each. **i & ii)** Unpaired t-test with Welch's correction. **(B, C, E)** Error bars show SD.

Fig. S9

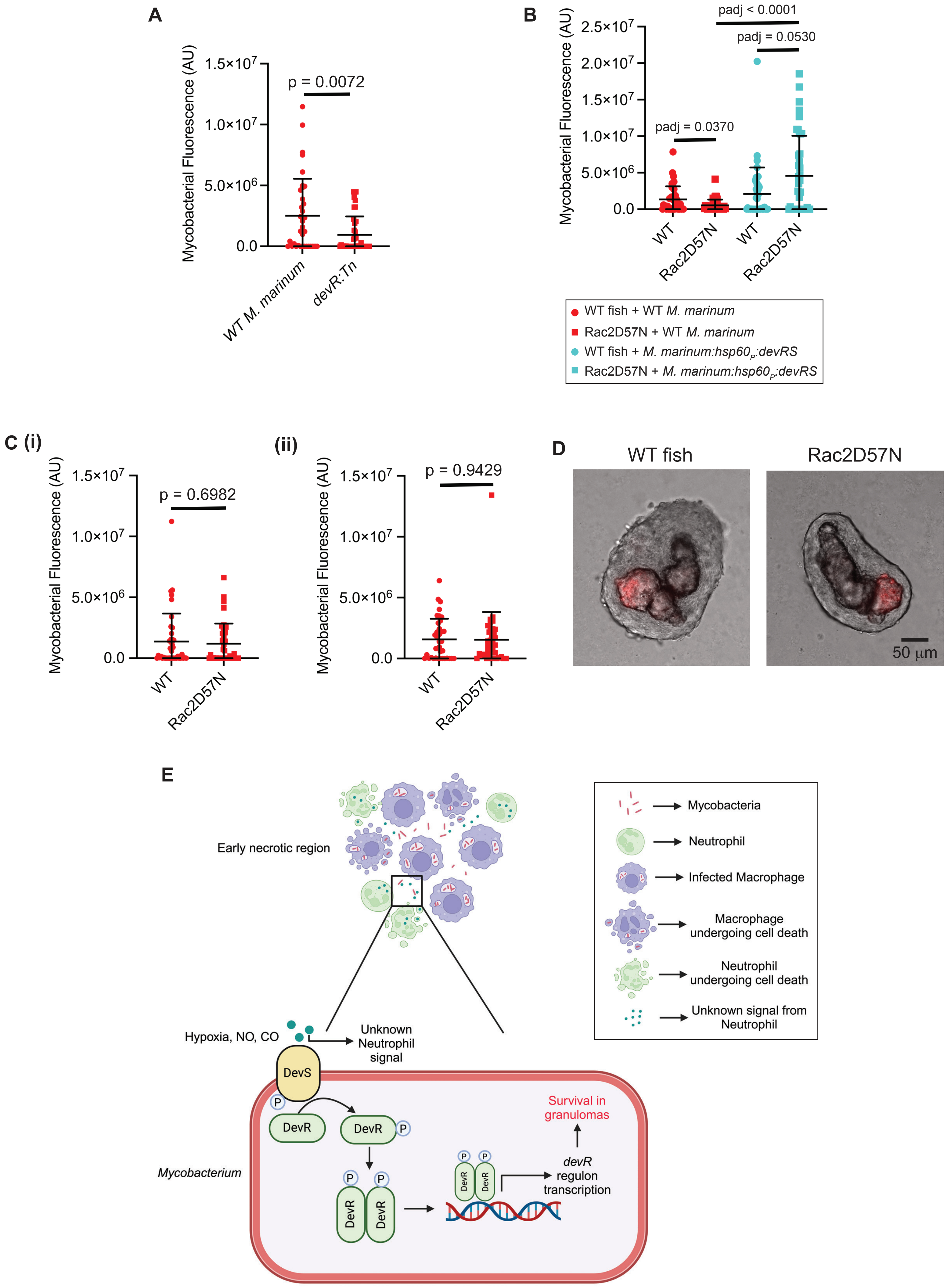
A
Mycobacterial Fluorescence (AU)
1.5×10^7
1.0×10^7
5.0×10^6
0.0
p = 0.0072
WT M. marinum
devR:Tn
B
Mycobacterial Fluorescence (AU)
2.5×10^7
2.0×10^7
1.5×10^7
1.0×10^7
5.0×10^6
0.0
padj < 0.0001
padj = 0.0530
padj = 0.0370
WT
Rac2D57N
WT
Rac2D57N
WT fish + WT M. marinum
Rac2D57N + WT M. marinum
WT fish + M. marinum:hsp60P:devRS
Rac2D57N + M. marinum:hsp60P:devRS
C (i)
Mycobacterial Fluorescence (AU)
1.5×10^7
1.0×10^7
5.0×10^6
0.0
p = 0.6982
WT
Rac2D57N
(ii)
Mycobacterial Fluorescence (AU)
1.5×10^7
1.0×10^7
5.0×10^6
0.0
p = 0.9429
WT
Rac2D57N
D
WT fish
Rac2D57N
50 μm
E
Early necrotic region
Mycobacteria
Neutrophil
Infected Macrophage
Macrophage undergoing cell death
Neutrophil undergoing cell death
Unknown signal from Neutrophil
Hypoxia, NO, CO
Unknown Neutrophil signal
DevS
P
DevR
DevR
P
Survival in granulomas
devR regulon transcription
Mycobacterium
DevR
DevR

Fig. S9. *devR* regulon plays a key role in neutrophil-driven modulation of mycobacterial burden. (A) Bacterial burden in WT *M. marinum* and *devR* mutant-infected granulomas dissected from WT fish, represented as mean arbitrary fluorescence units (AU). For WT *M. marinum,* n = 36 granulomas; for *devR* mutant, n = 41 granulomas. Granulomas were obtained from 3 animals per group. Unpaired t-test with Welch's correction. The data show the second independent experiment corresponding to Fig. 4G. **(B)** Granuloma bacterial burden in WT and Rac2D57N fish infected with either WT *M. marinum* (red data points) or *M. marinum* overexpressing *devR* & *devS* (cyan data points), represented as mean arbitrary fluorescence units (AU). For WT *M. marinum* infection: n = 39 granulomas each from WT and Rac2D57N fish. For *M. marinum devRS* overexpressor infection: n = 40 granulomas from WT fish and 41 granulomas from Rac2D57N fish. Brown-Forsythe and Welch ANOVA tests, followed by Dunnett's T3 multiple comparisons test, were used. Granulomas were obtained from 3 animals per group. The data show the second independent experiment corresponding to Fig. 4I. **(C) i & ii:** Granuloma bacterial burden in WT and Rac2D57N fish infected with *devR* mutant, represented as mean arbitrary fluorescence units (AU). **i & ii** are independent biological replicates. **(i)** n = 40 granulomas each from WT & Rac2D57N fish. **(ii)** n = 37 granulomas each from WT & Rac2D57N fish. Granulomas were obtained from 4 animals per group. **(i)** Unpaired t-test with Welch's correction, **(ii)** Unpaired t-test. **(D)** Representative images showing the burden of *devR* mutant in granulomas dissected from infected WT and Rac2D57N fish. Scale bar, 50 µm. **(A-D)** Granulomas were dissected from 14 dpi animals. **(A, B, C)** Each data point represents mycobacterial fluorescence measured in a single granuloma. Error bars indicate SD. **(E)** Model describing the role of neutrophils in promoting granuloma mycobacterial survival through the regulation of the *devR* regulon. Neutrophils release an unknown signal, either through diffusion or cell death in the early necrotic region. This signal is sensed by extracellular mycobacteria via the membrane-associated gas sensor, histidine protein kinase DevS, part of a two-component regulatory system. DevS then undergoes autophosphorylation, leading to the phosphorylation and activation of the response regulator DevR, a DNA-binding protein that drives the expression of the *devR* regulon. This regulon enhances mycobacterial survival under the hostile conditions present in necrotic granulomas, such as hypoxia and NO stress. The unknown signal may act cumulatively with known DevS signals, like hypoxia and NO, to upregulate the *devR* regulon. Created with BioRender.com.

Fig. S10

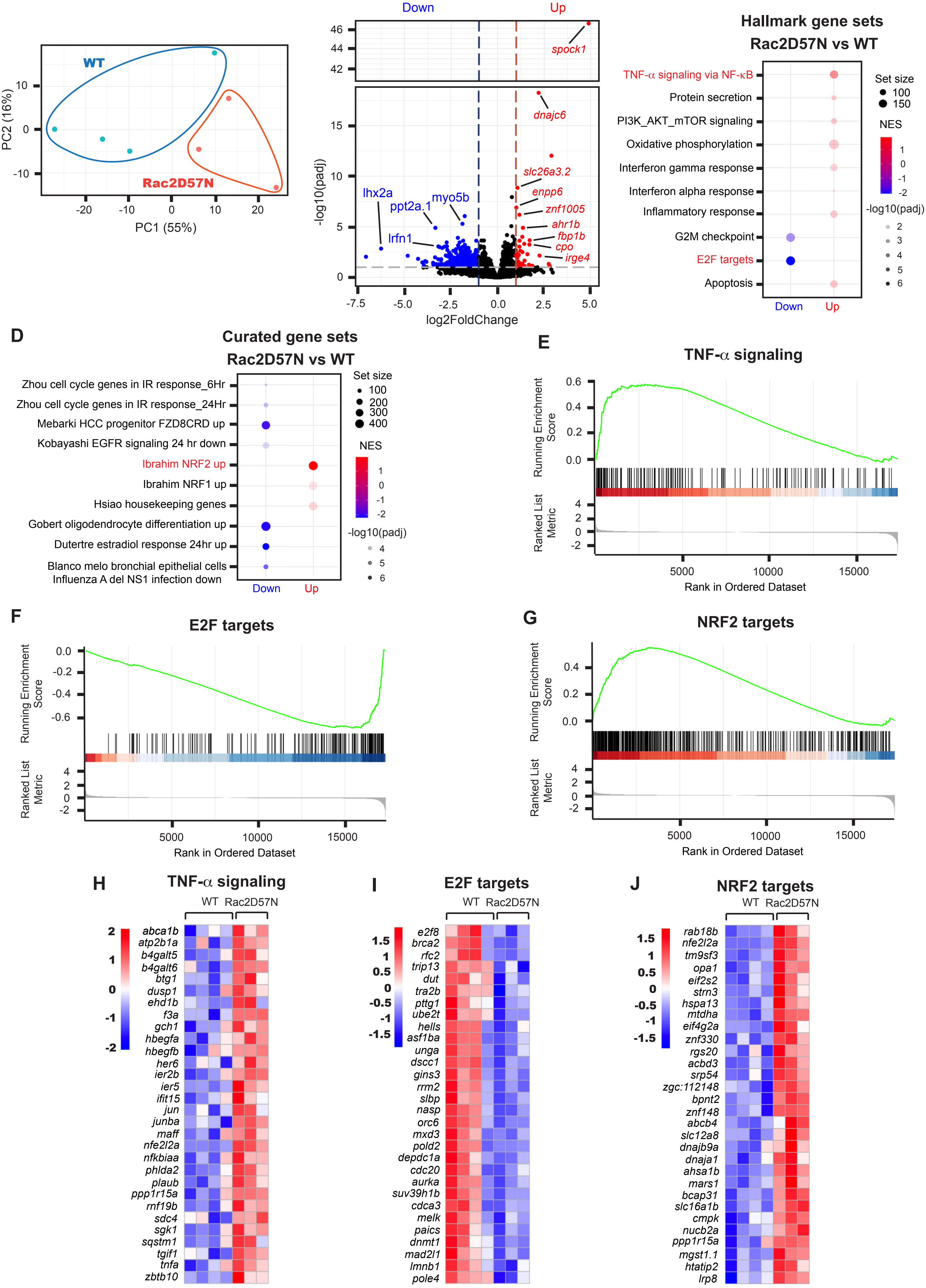
A
WT
Rac2D57N
PC2 (16%)
PC1 (55%)
B
Rac2D57N vs WT
Down
Up
spock1
dnajc6
slc26a3.2
enpp6
znf1005
ahr1b
fbp1b
cpo
irge4
lhx2a
ppt2a.1
myo5b
lrfn1
-log10(padj)
log2FoldChange
C
Hallmark gene sets
Rac2D57N vs WT
TNF-α signaling via NF-κB
Protein secretion
PI3K_AKT_mTOR signaling
Oxidative phosphorylation
Interferon gamma response
Interferon alpha response
Inflammatory response
G2M checkpoint
E2F targets
Apoptosis
Set size
NES
-log10(padj)
D
Curated gene sets
Rac2D57N vs WT
Zhou cell cycle genes in IR response_6Hr
Zhou cell cycle genes in IR response_24Hr
Mebarki HCC progenitor FZD8CRD up
Kobayashi EGFR signaling 24 hr down
Ibrahim NRF2 up
Ibrahim NRF1 up
Hsiao housekeeping genes
Gobert oligodendrocyte differentiation up
Dutertre estradiol response 24hr up
Blanco melo bronchial epithelial cells
Influenza A del NS1 infection down
E
TNF-α signaling
Running Enrichment Score
Ranked List Metric
Rank in Ordered Dataset
F
E2F targets
G
NRF2 targets
H
TNF-α signaling
WT
Rac2D57N
abca1b
atp2b1a
b4galt5
b4galt6
btg1
dusp1
ehd1b
f3a
gch1
hbegfa
hbegfb
her6
ier2b
ier5
ifit15
jun
junba
maff
nfe2l2a
nfkbiaa
phlda2
plaub
ppp1r15a
rnf19b
sdc4
sgk1
sqstm1
tgif1
tnfa
zbtb10
I
E2F targets
e2f8
brca2
rfc2
trip13
dut
tra2b
pttg1
ube2t
hells
asf1ba
unga
dscc1
gins3
rrm2
slbp
nasp
orc6
mxd3
pold2
depdc1a
cdc20
aurka
suv39h1b
cdca3
melk
paics
dnmt1
mad2l1
lmnb1
pole4
J
NRF2 targets
rab18b
nfe2l2a
tm9sf3
opa1
eif2s2
strn3
hspa13
mtdha
eif4g2a
znf330
rgs20
acbd3
srp54
zgc:112148
bpnt2
znf148
abcb4
slc12a8
dnajb9a
dnaja1
ahsa1b
mars1
bcap31
slc16a1b
cmpk
nucb2a
ppp1r15a
mgst1.1
htatip2
lrp8

Fig. S10. Identification of host genes differentially regulated in neutrophil-deficient granulomas. (A) Principal-component analysis (PCA) of the host transcriptome from *M. marinum*-infected wild-type and Rac2D57N granulomas. (B) Volcano plot showing differential expression of the host genes in Rac2D57N vs WT granulomas. The horizontal dotted line indicates a p_{adj} threshold of 0.1, whereas the vertical dotted lines represent log2 fold change thresholds of -1 and 1 respectively. **(C)** Bubble plot based on Gene Set Enrichment Analysis (GSEA) of the host transcriptome, showing Hallmark gene sets from the Molecular Signatures Database (MSigDB) that are up or downregulated in Rac2D57N vs WT granulomas. **(D)** GSEA bubble plot of the host transcriptome, showing the top ten Curated gene sets from MSigDB that are significantly up or downregulated in Rac2D57N vs WT granulomas. **(C, D)** The size of each circle represents the weighted number of genes involved in the term. The color and intensity of the bubbles represent the enrichment score and -log10 (p_{adj}) values, respectively. Gene sets relevant to this study are marked in red. **(E, F, G)** Enrichment score (ES) plots for the gene sets corresponding to TNF- α signaling, E2F targets, and NRF2 targets, as highlighted in C & D. Positive and negative enrichment scores indicate gene sets that are over-represented among the most upregulated or downregulated genes in Rac2D57N granulomas. Black vertical bars represent individual genes within a gene set, with their position reflecting each gene's contribution to the overall enrichment score. Genes appearing at or before the enrichment score maximum (**E, G**) and at or after the enrichment minimum (**F**) contribute to the enrichment signal. The bottom portion of the plot shows the value of the ranking metric along the list of ranked genes. **(H, I, J)** Heatmaps showing relative expression levels for the host genes associated with TNF- α signaling, E2F targets, and NRF2 targets from WT and Rac2D57N granulomas.

Fig. S11

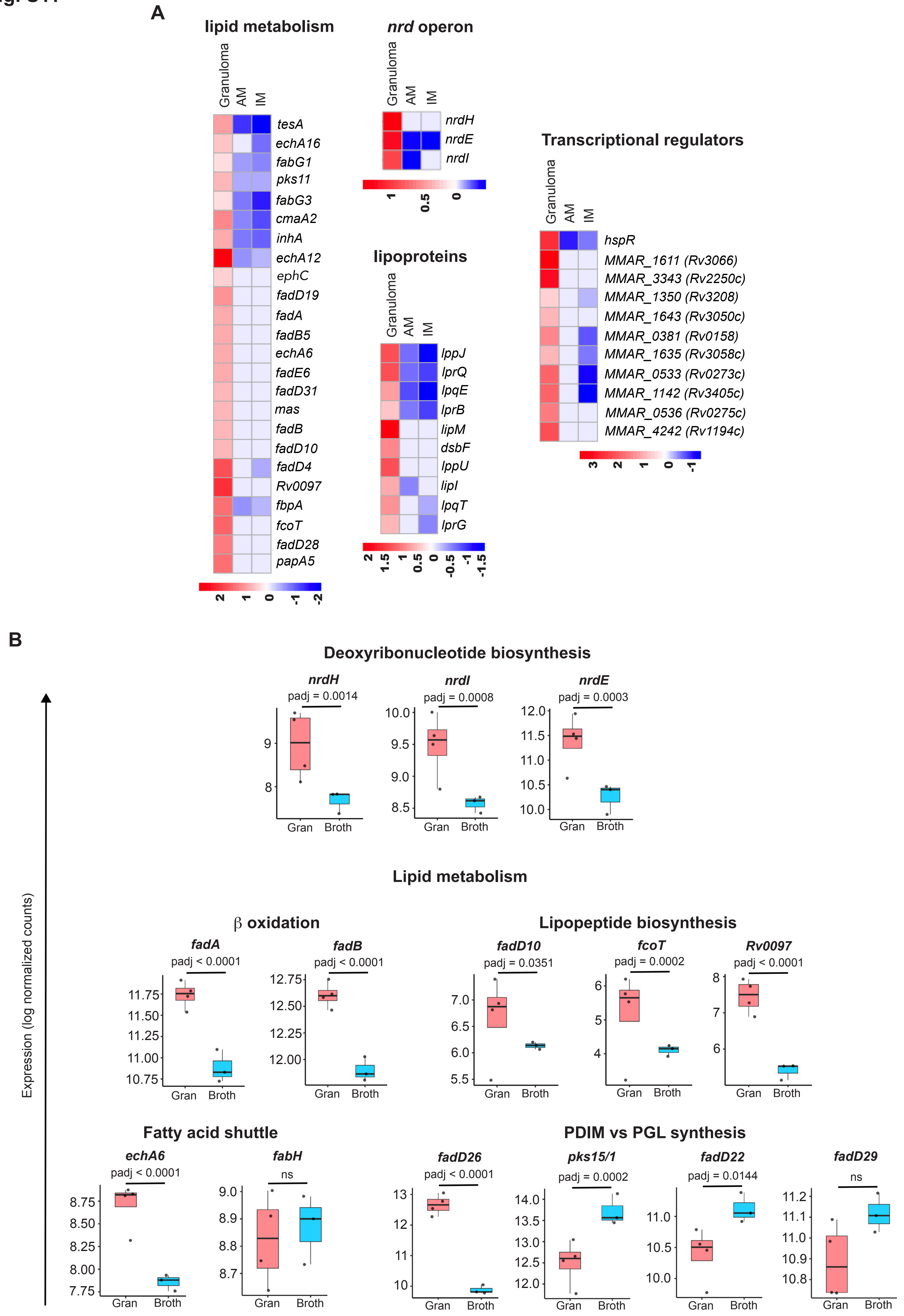

A
lipid metabolism
Granuloma
AM
IM
tesA
echA16
fabG1
pks11
fabG3
cmaA2
inhA
echA12
ephC
fadD19
fadA
fadB5
echA6
fadE6
fadD31
mas
fadB
fadD10
fadD4
Rv0097
fbpA
fcoT
fadD28
papA5
2 1 0 -1 -2
nrd operon
nrdH
nrdE
nrdI
1 0.5 0
lipoproteins
lppJ
lprQ
lpqE
lprB
lipM
dsbF
lppU
lipI
lpqT
lprG
2 1.5 1 0.5 0 -0.5 -1 -1.5
Transcriptional regulators
hspR
MMAR_1611 (Rv3066)
MMAR_3343 (Rv2250c)
MMAR_1350 (Rv3208)
MMAR_1643 (Rv3050c)
MMAR_0381 (Rv0158)
MMAR_1635 (Rv3058c)
MMAR_0533 (Rv0273c)
MMAR_1142 (Rv3405c)
MMAR_0536 (Rv0275c)
MMAR_4242 (Rv1194c)
3 2 1 0 -1
B
Expression (log normalized counts)
Deoxyribonucleotide biosynthesis
nrdH
padj = 0.0014
nrdI
padj = 0.0008
nrdE
padj = 0.0003
Lipid metabolism
β oxidation
fadA
padj < 0.0001
fadB
padj < 0.0001
Lipopeptide biosynthesis
fadD10
padj = 0.0351
fcoT
padj = 0.0002
Rv0097
padj < 0.0001
Fatty acid shuttle
echA6
padj < 0.0001
fabH
ns
PDIM vs PGL synthesis
fadD26
padj < 0.0001
pks15/1
padj = 0.0002
fadD22
padj = 0.0144
fadD29
ns
Gran
Broth

Fig. S11. Identification of additional mycobacterial transcripts enriched in necrotic wild-type granulomas. (A) Heatmaps showing mycobacterial genes specifically upregulated in zebrafish necrotic granulomas vs in vivo mouse macrophages, related to lipid metabolism, deoxyribonucleotide biosynthesis (*nrd* operon), lipoproteins, and transcriptional regulators. **(B)** Box plots showing the median log normalized counts of representative transcripts from **(A)** in WT granulomas and broth culture. Each data point represents the log normalized count of the respective transcript from an individual experiment. For the fatty acid shuttle, *M. marinum* shows upregulation of *echA6* but not the functional analog *fabH* in granulomas suggesting a key role for *echA6* in fatty acid transfer and cell envelope lipid biosynthesis in this niche. In the interrelated PDIM-PGL biosynthesis pathways, specific upregulation of the *fadD26* compared to genes involved in PGL precursor synthesis suggests a shift in the balance towards PDIM production in granulomas. Benjamini-Hochberg (BH) correction was used to determine the adjusted p-values.

Supplementary table legends

Table S1. ScRNA-seq data showing the proportion of granuloma neutrophil subgroups and differentially expressed genes within each subgroup. Data corresponds to Fig. S4

Table S2. Gene Ontology (GO) terms enriched in each neutrophil subgroup. Data corresponds to Fig. S4

Table S3. Replicate ScRNA-seq data showing the proportion of granuloma neutrophil subgroups and differentially expressed genes within each subgroup. Data corresponds to Fig. S5.

Table S4. Sequences of zebrafish and *M. marinum* rRNA depletion probes used in the dual RNA-seq experiment in this study

Table S5. RNA-seq data showing differentially expressed *M. marinum* genes in Rac2D57N vs WT granulomas

Table S6. RNA-seq data showing differentially expressed zebrafish genes in Rac2D57N vs WT granulomas. Separate tabs present Gene Set Enrichment Analysis (GSEA) data for the zebrafish transcriptome, including Hallmark and Curated (C2) gene sets from the Molecular Signatures Database (MSigDB) that are upregulated or downregulated in Rac2D57N vs WT granulomas. Additionally, expression data for the host genes related to key gene sets, such as TNF- α signaling, E2F targets, and NRF2 targets are provided separately.

Table S7. RNA-seq data showing differentially expressed *M. marinum* genes in WT granulomas vs broth culture.

Table S8. Expression data for mycobacterial genes commonly upregulated in zebrafish necrotic granulomas and in vivo mouse macrophages.

Table S9. Expression data for mycobacterial genes specifically upregulated in zebrafish necrotic granulomas compared to in vivo mouse macrophages.

Table S10. Lineage-specific non-synonymous and synonymous mutations identified in the top 50 granuloma-specific genes. Genes with multiple non-synonymous mutations are highlighted in green. The Sequence Read Archive (SRA) list of 69 clinical isolates is provided in a separate tab.

Supplementary movie legends

Movie S1. Split-screen video showing neutrophils (green) tracked over 230 minutes in *M. marinum* (red) infected type I and extra-necrotic region of type II granuloma explants. Frames were captured at 10-minute intervals. Individual tracks are labeled in white, magenta, and red. Maximum intensity projections, 100 μm. Scale bar, 50 μm. The video corresponds to **Fig. 1E**.

Movie S2. Neutrophil migration between sub-compartments of a type II granuloma. The movement of two neutrophils (red and white arrows), initially found near the necrotic core, toward the *M. marinum* containing extra-necrotic region (white box) was tracked over 240 mins. Frames were

captured at 10-minute intervals. Maximum intensity projection, 100 µm. Scale bar, 50 µm. The video corresponds to **Fig. S1**.

Movie S3. Neutrophil viability in *M. marinum* infected type I and extra-necrotic region (white box) of type II granulomas. Granulomas were observed for 790 minutes, with individual frames captured at 10-minute intervals. Maximum intensity projections, 100 µm. Scale bar, 50 µm. The video corresponds to **Fig. 1G**.

Movie S4. Video showing the viability of an uninfected neutrophil (red arrow) and an infected neutrophil (white arrow) in the *M. marinum* containing extra-necrotic region of a type II granuloma. Individual frames were acquired at 10-minute intervals. 100 µm maximum intensity projection. Scale bar, 50 µm. The video corresponds to **Fig. S2**.

Movie S5. Split-screen video showing neutrophil (green) and macrophage (red) viability in *M. marinum* (magenta) infected type I and extra-necrotic region (white box) of type II granulomas. Granulomas were observed for 780 minutes, with frames captured at 10-minute intervals. 100 µm maximum intensity projections. Scale bar, 50 µm. The video corresponds to **Fig. S3A**.

Movie S6. Interaction between a dying neutrophil (red arrow) and an *M. marinum* (magenta) infected macrophage (white arrow) in the extra-necrotic region of a type II granuloma. Neutrophils are labeled green and macrophages in red. The yellow arrow indicates *M. marinum* released from the infected macrophage following its interaction with the neutrophil. Frames were captured at 10-minute intervals. Maximum intensity projection, 100 µm. Scale bar, 25 µm. The video corresponds to **Fig. S3B**.

Movie S7. Neutrophils (green) tracked over 230 minutes in *M. marinum* (red) infected granuloma explants, 5 hours post-treatment with DMSO/duvelisib. Frames were captured at 10-minute intervals. Individual tracks are labeled in white, magenta, and red. 100 µm maximum intensity projections. Scale bar, 50 µm. The video corresponds to **Fig. 2C**.
